# Kidney Donor Risk Index and Cardiovascular Complications in a Long-Term Follow-Up Observation

**DOI:** 10.3390/jcm14072346

**Published:** 2025-03-29

**Authors:** Agata Kujawa-Szewieczek, Natalia Słabiak-Błaż, Aureliusz Kolonko, Andrzej Więcek, Grzegorz Piecha

**Affiliations:** Department of Nephrology, Transplantation and Internal Medicine, Medical University of Silesia, Francuska 20/24, 40-027 Katowice, Poland; nataliablaz@gazeta.pl (N.S.-B.); uryniusz@wp.pl (A.K.); awiecek@sum.edu.pl (A.W.); g.piecha@outlook.com (G.P.)

**Keywords:** kidney transplantation, Kidney Donor Profile Index, graft function, cardiovascular disease, prognosis

## Abstract

**Background**: The suitability of the Kidney Donor Risk Index (KDRI) has not been fully validated in the European population. The aim of this study was to evaluate the value of the KDRI in predicting kidney graft function and cardiovascular events (CVEs) in a Polish cohort of kidney transplant recipients (KTRs). **Methods**: In this retrospective study kidney graft function and CVEs were analyzed among 1420 patients transplanted between 1999 and 2017 and followed until 2021. The KDRI was calculated according to the formula proposed by Rao. Patients were assigned into quartiles (Qs) of KDRI values. **Results**: Patients in Q4 were older, with higher BMI, longer cold ischemia time (CIT), and a greater rate of ischemic heart disease at the transplantation. The KDRI value determined both early and long-term graft function. During a median follow-up period of 91 months, at least one cardiovascular event was noted in 227 (16.0%) kidney transplant recipients. There was a significant increasing trend for the occurrence of post-transplant CV complications along the consecutive KDRI quartiles (χ^2^ = 7.3; *p* < 0.01) among kidney transplant patients younger than 50 years at the time of transplantation. **Conclusions**: The KDRI is an adequate prognostic tool also for the European population. Despite the KDRI not being used for allocation in Poland we found that kidneys with a higher KDRI are allocated to recipients with worse survival prognosis. The quality of kidneys from a deceased donor may be related to the occurrence of post-transplant cardiovascular complications in recipients younger than 50 years at the transplantation, including those without history of comorbidities such as diabetes or cardiovascular disease.

## 1. Introduction

Kidney transplantation (KT) is actually a promoted method of renal replacement therapy. In comparison to long-term dialysis treatment, a successful KT increases survival and improves the quality of life of patients with stage 5 chronic kidney disease (CKD) [1]. Although short-term graft and transplant recipients’ survival has been improved in recent years, the long-term outcomes have not changed significantly. The leading cause of graft loss is still death with a functioning graft, with cardiovascular diseases (CVDs) as a main cause of hospitalization and death after successful KT [2]. There are several traditional and specific for post-transplant period risk factors for the development of CVDs [2]. A type of donor and, indirectly, quality of the graft may potentially be a predictor of cardiovascular events (CVEs) after kidney transplantation [3,4].

One of the major challenges as well as the main limitation of KT remains donor organ shortage. Despite an increasing number of patients with CKD on the waiting list, a high discard rate is still observed [5]. In an attempt to increase the donor pool, high attention was paid to improve the use of marginal deceased donor kidneys. On the other hand, a good quality of donated kidney is crucial in determining acceptable long-term transplant outcomes. The Kidney Donor Risk Index (KDRI) was proposed by Rao et al. [6] as a new clinical donor quality scoring system to facilitate the decision whether to accept or to decline donated organs in United States (US). In the most commonly used form, proposed by the Organ Procurement and Transplantation Network (OPTN) in 2014, the KDRI consists of 10 variables, including demographics and medical history of the donor as well as factors related to the donor death. This donor risk index allows dividing the donor population into quintiles based on their KDRI. It has been proved that higher KDRI values were associated with shorter graft survival as compared to kidneys with a lower KDRI [6,7]. Moreover, this correlation was similar among both expanded (ECDs) and standard criteria donors (SCDs), suggesting that KDRI implementation can improve the utilization of marginal donor kidneys [8]. Following this, the Kidney Donor Profile Index (KDPI), derived by numerically mapping the KDRI from a relative risk scale to a percentile, was calculated and in 2014 a new kidney “longevity matching” allocation system was implemented in the US. According to this assumption kidneys of the best quality are preferentially transplanted into recipients with estimated better post-transplant survival prognosis. It should be emphasized that applicability of the KDRI score has been assessed for the American population [6] and its usage in the European, including Polish, cohort has not yet been fully validated. The aim of our study was to evaluate the association between the KDRI and recipient CVEs as well as kidney graft function in a Polish transplant cohort.

## 2. Materials and Methods

### 2.1. Study Group

This retrospective single-center cohort study included 1600 adult patients who received KT from January 1999 to December 2017. Living donor patients (*n* = 48), simultaneous pancreas–kidney (SPK, *n* = 92) transplant recipients, and patients with missing donor data (*n* = 40) were excluded. For the remaining 1420 patients, the value of the KDRI was calculated and patients were divided into 4 study groups according to the KDRI quartiles.

The initial triple immunosuppression protocol was based on cyclosporine A or tacrolimus, mycophenolate mofetil or mycophenolate sodium, or everolimus and steroids. Pre-transplant CVEs were defined as stroke, myocardial infarction, coronary artery bypass grafting (CABG), or stenting performed for an indication other than acute myocardial infarction. Acute myocardial infarction was diagnosed according to the current definition. Each case where a coronal artery stenting procedure was performed, or where there was an indication for CABG following the diagnosis of acute myocardial infarction, was classified as a single cardiovascular episode and was counted as acute myocardial infarction. In other cases, the qualification for coronal artery stenting or CABG was not preceded by a diagnosis (in accordance with the definition) of acute myocardial infarction. If, after coronary angiography, the patient was qualified for CABG, the complication was also counted as only one event—in this case, CABG.

### 2.2. KDRI Assessment

The KDRI was calculated retrospectively according to the formula proposed by Rao et al. and modified by the OPTN [6], using 10 variables, including information of donor demographics and medical history (age, height, weight, ethnicity, arterial hypertension, diabetes, hepatitis C status) as well as elements related to donor death (cause of death, brain or cardiac death, and terminal serum creatinine). All transplanted patients enrolled in this study were divided into quartiles (Qs) according to the KDRI value.

### 2.3. Patient and Graft Outcomes

Immediate graft function (IGF) was characterized by a serum creatinine level (S_cr_) < 3 mg/dL at the post-operative day (POD) 3 and slow graft function (SGF) as S_Cr_ > 3 mg/dL at POD 3. Delayed graft function (DGF) was diagnosed in patients who required dialysis therapy during the first week after KT. The long-term transplant outcomes were observed until December 2021 or to the time of graft loss. Kidney graft function was evaluated yearly. Based on S_cr_ values, an estimated glomerular filtration rate (eGFR) was calculated using the Modification of Diet in Renal Disease (MDRD) formula. Post-transplant CVEs included stroke, myocardial infarction, CABG, or stenting performed for an indication other than acute myocardial infarction. Common post-transplant complications, including acute rejection (AR) episodes, post-transplant diabetes mellitus (PTDM), and cytomegalovirus (CMV) infections, were also noted.

### 2.4. Statistical Analysis

Statistical analyses were performed using STATISTICA 13.3 PL for Windows (Tibco Inc., Palo Alto, CA, USA) and MedCalc v19.2.1 (MedCalc Software, Mariakerke, Belgium). Values were presented as means with a 95% confidence interval, medians with Q1–Q3 values, or frequencies. The main comparison was performed between four groups of patients based on the KDRI quartiles, using the ANOVA test (for quantitative variables) or the χ^2^ test (for qualitative variables). Variables with non-normal distribution were compared using the Kruskal–Wallis test. The comparisons between particular groups were performed using the Mann–Whitney U test. Correlation analyses were performed using the Spearman rank test. Stepwise multiple regression analysis was performed for the risk of post-transplant CVEs as the dependent variable with recipients’ age, body mass index (BMI), pre-transplant ischemic heart disease, the use of induction therapy, and PTDM as potential independent variables. Similar analysis in a subset of patients younger than 50 years at the time of transplantation was also performed, with recipients’ age, pre-transplant ischemic heart disease, PTDM, and KDRI value as potential independent variables. The regression models were built based on the results of univariate analyses, with *p* < 0.1 as the inclusion criterion. For all analyses, a *p*-value below 0.05 was considered statistically significant.

## 3. Results

### 3.1. Study Group

Table 1 shows the clinical characteristics of kidney transplant patients divided into four groups based on KDRI values. At transplantation, patients in Q4 were significantly older than the rest of the study participants, as the national kidney allocation system according to the “old-to-old” policy promotes the transplantation of organs procured from donors >65 years to the recipients >60 years. BMI values and cold ischemia time (CIT) in Q4 were also significantly higher than in Q1–3. There were significantly more patients receiving induction therapy (36.4%) in Q4 than in the other study groups (26.5% in Q1 vs. 27.5% in Q2 vs. 26.9% in Q3; *p* < 0.05). Additionally, there were statistically significant differences between KDRI quartiles in relation to the number of antihypertensive drugs prior to transplantation and the percentage of second and third transplantations in the past (Table 1). There were no significant differences in the pre-transplant dialysis period, residual diuresis, HLA mismatches, and pre-transplant CVEs between study groups, except for the rate of ischemic heart disease at the transplantation, which was significantly greater in Q4 than in Q1 and Q2.

In the whole study group, the causes of end-stage kidney disease (ESKD) were as follows: glomerulonephritis (45.6%), diabetic nephropathy (7.0%), pyelonephritis (10.8%), ADPKD (10.6%), hypertensive nephropathy (10.8%), other (3.4%), and unknown (11.8%), and they did not differ between kidney transplant patients allocated to the four quartiles. Importantly, patients in Q1 significantly more often received the initial immunosuppressive regimen based on cyclosporine A than tacrolimus in comparison with the other study groups (Table 1).

During the follow-up period, the frequency of AR episodes and CMV infections was similar in all quartiles, but there was greater occurrence of PTDM in patients from Q3 and Q4 (Table 1). Importantly, the prospective monitoring of medical events during the long-term observation period was almost complete, as only seven patients (0.5%) were lost to follow-up.

### 3.2. KDRI Values

The median KDRI in the whole study group was 1.092 (Q1–Q3: 0.881–1.330), with a range of 0.583–4.734. The median KDRI values in the consecutive study groups were as follows: Q1 0.795 (0.736–0.836), Q2 0.979 (0.935–1.023), Q3 1.200 (1.150–1.255), and Q4 1.543 (1.420–1.811). The median donor age was 46 (34–53) years, and the median BMI was 24.7 (23.0–26.6) kg/m^2^. The donor cohort was exclusively Caucasian and all donations were performed after diagnosis of the brain death. Twenty-one percent of donors were hypertensive and 23.2% had a last serum creatinine test prior to organ procurement > 1.5 mg/dL. Additionally, there were only six transplantations using organs from donors with diabetes mellitus (0.4%). Finally, 122 organs were procured from donors with the presence of anti-HCV antibodies (8.5%) and the absence of HCV viremia at the time of donation.

### 3.3. Early and Long-Term Kidney Graft Function

There was no difference in the occurrence of primary kidney graft non-function among study groups. Across the increasing KDRI quartiles, a significantly less frequent occurrence of IGF was observed; instead, there was an increasing trend for SGF and DGF incidence, with their highest rate in Q4 (Table 2). In a long-term observation, the highest eGFR values were noted in Q1 group and the lowest in Q4 group.

Significant associations were found between KDRI value and eGFR at 3 months (R = 0.341), 12 months (R = 0.359), and 60 months (R = 0.349), with all *p* < 0.001.

### 3.4. KDRI Values and Cardiovascular Complications

In the whole study group, during a median follow-up period of 91 (56–136) months, at least one CVE was noted in 227 (16.0%) kidney transplant recipients. In order to exclude the influence of preexisting and undiagnosed CAD and to assess the impact of the KDRI on the incidence of CVEs, all cardiovascular events occurring within the first year after transplantation (*n* = 31) were excluded from the main analysis in our study. Thus, 195 (13.7%) first post-transplant cardiovascular episodes were analyzed, which occurred at a median of 71 (40–105) months after the last KT, with a range of 13–224 months. The median time to first CVE did not differ between study groups (*p* = 0.2). There were 104 myocardial infarctions, 61 strokes, 23 coronal stentings, and seven CABG procedures. The structure of cardio- and cerebrovascular events did not differ between study groups (*p* = 0.68).

A modest association between KDRI value and recipients’ age was noted (R = 0.215, *p* < 0.001). In the whole study cohort, the percentage of patients with post-transplant CVEs was significantly lower in Q1 [36 (10.1%)] than in Q3 [56 (15.7%); *p* < 0.05], whereas the difference between Q1 and the other quartiles did not reach the statistical significance [Q2: 52 (14.6%); *p* = 0.07, Q4: 51 (14.5%); *p* = 0.09]. However, among kidney transplant patients younger than 50 years at the time of transplantation (*n* = 754), there was a significant trend for increasing the occurrence of post-transplant CV complications along the consecutive KDRI quartiles (χ^2^ = 7.3; *p* < 0.01), with the frequency of CVEs across the study groups as follows: Q1 13/205, Q2 23/179, Q3 24/166, and Q4 21/123.

The results of univariate logistic regression analyses for factors increasing the risk of post-transplant CVEs in the whole study cohort and in a group of patients younger than 50 years at the time of transplantation are shown in Table 3.

In the whole study cohort, stepwise multivariate regression analysis revealed that recipient age (r_partial_ = 0.106, *p* < 0.001), pre-transplant ischemic heart disease (r_partial_ = 0.077, *p* < 0.01), and PTDM (r_partial_ = 0.086, *p* < 0.01) independently increased the risk for post-transplant CVEs, whereas the lack of induction therapy (r_partial_ = −0.074, *p* < 0.01) decreased that risk, and BMI was not included in the final model. In a separate stepwise multivariate regression analysis in a subset of recipients younger than 50 years at the time of transplantation, only recipient age (r_partial_ = 0.200, *p* < 0.001), pre-transplant ischemic heart disease (r_partial_ = 0.103, *p* < 0.01), and PTDM (r_partial_ = 0.090, *p* < 0.05), but not KDRI value, independently influenced the risk of post-transplant cardiovascular complications.

## 4. Discussion

This study is so far one of few publications evaluating the assessment of the Kidney Donor Risk Index in a European cohort and the first one concerning the association between the KDRI and cardiovascular complications in a long-term follow-up. The results obtained in our study suggest that the KDRI may be a valuable prognostic tool for early and long-term outcomes after KT in the Polish cohort, with a higher risk of DGF or SGF in the early post-transplantation period and a lower eGFR in a long-term observation among the highest KDRI values. The analysis of cardiovascular complications in the follow-up period revealed a significant trend for increasing the occurrence of post-transplant CV complications along the consecutive KDRI quartiles only in the subgroup of patients younger than 50 years at the time of transplantation. Additionally, the lowest percentage of patients with post-transplant CVEs was identified among the Q1 cohort.

An appropriate evaluation of clinical donor-related parameters and a proper organ allocation can significantly improve the post-transplant outcomes. Moreover, in the situation of global organ shortages, a precise qualification of kidneys from marginal donors may significantly increase the donor pool, particularly for the elderly patients on the waiting list. The KDRI is used to predict the outcomes of kidney transplantation from a deceased donor in the US since 2014. So far, the validation of the KDRI or KDPI in a European cohort has been analyzed in several studies [9,10,11,12,13,14]. Implementation of the KDRI at the time of donation led to an increase of 5.4% in accepted donor kidneys at the Antwerp University Hospital [9]. Moreover, the median KDRI of all transplanted kidneys during this prospective study was significantly higher (0.97) than the median KDRI (0.85) during a previous retrospective observation in this population [9]. Lehner et al. [10] have shown that the KDPI was an independent predictor of lower graft survival in the German population. Despite a higher relative risk of graft loss, a 10-year death-censored graft survival in the group of >85% KDPI was estimated as high as 62%. Similarly, a positive predictive power of the KDPI in terms of 1-year eGFR and death-censored graft survival was presented in another German kidney transplant cohort [11]. The KDRI was also a valuable tool for estimating graft survival and facilitating the decision of donation from the expanded criteria donors (ECDs) pool in Spain [12]. The authors reported that a 0.1-point increase in the KDRI score led to a higher (by 9.5% per year) risk of graft failure [12]. In a recent study, Pippias et al. [13] analyzed time trends in the KDRI between 2005 and 2015 among adult kidney transplant recipients in seven European countries. Although the median crude KDRI increased from 1.31 to 1.47, the patient and graft survival did not change significantly during these years. Moreover, within each time cohort, patient and graft survival was higher at lower KDRIs.

In the present study, we showed a positive correlation between the recipient’s age and the KDRI value. According to Dahmen et al. [11], high KDPI values appeared to be mainly dependent on the donor’s age. Similarly, in our observation kidneys with the highest KDRI values were allocated to older recipients as a consequence of an “old-to-old” allocation program. Moreover, recipients of high-KDRI kidneys had a higher BMI and greater rate of ischemic heart disease prior to transplantation. We did not find an association between KDRI value and HLA mismatches; nonetheless, there were significantly more patients receiving induction therapy (36.4%) and an initial immunosuppressive regimen based on tacrolimus than cyclosporine A in the highest-KDRI cohort. Finally, the longest cold ischemia time was observed among recipients of higher-KDRI donor kidneys. Therefore, it can be assumed that lower-quality kidneys in our cohort were allocated to patients with the highest burden of comorbidity and thus shorter life expectancy.

According to published data DGF is associated with a worse allograft and patient survival [15]. The incidence of DGF has increased over the past decades partly as a result of the expanding use of kidneys from marginal donors [16]. Analysis of the graft function in the early post-transplant period in our study group revealed an association between KDRI value and risk of DGF or SGF, with their highest incidence (38% and 48%, respectively) in the KDRI Q4 group. Parallel to this observation, other authors noted that the difference between the time to equal survival in recipients with and without DGF was similar in kidneys with a KDPI of 20% to 60% (250–279 days), whereas the difference in time to equal survival was greatest among kidneys with a KDPI > 80% (809 days) [17]. Moreover, in the study by Salguero et al. [18], the presence of DGF was identified as a significant risk factor for graft loss, particularly among grafts from donors with a higher KDRI (KDRI > 1). Our observation is indirectly in line also with a previously published study by Lehner et al. [10]. Although the authors showed that DGF occurred more frequently in the higher-KDPI groups, no further increase in DGF rates was seen in the highest KDPI categories. Darema et al. reported that in a Greek cohort DGF was associated with a significantly greater KDRI value only in standard-criteria donor transplantations [19]. One can assume that other factors besides donor characteristics are equally important in the development of DGF. As we mentioned above, recipients of organs with a higher KDRI were characterized by a longer CIT in the present study.

As expected, the cohort with the highest-KDRI kidneys showed significantly worse long-term graft excretory function. This significant association was found between KDRI value and eGFR throughout the entire follow-up period, with the difference in eGFR between the KDRI in Q1 and Q4 at 60 months post-transplant up to 22 mL/min/1.73 m^2^. Nevertheless, stable median eGFR levels in the highest-KDRI kidneys, with a slight slop of 1.1 mL over 5 years (from 42.6 to 43.7 mL/min/1.73 m^2^), is in our opinion a quite acceptable result. In a recently published analysis, the median crude KDRI in a European transplant population increased from 2005 to 2015 by 1.3% per year [13]. Despite the worse quality of kidneys from deceased donors, five-year patient and graft survival probabilities remained unchanged over time [13].

Cardiovascular events remain a common complication after KT and account for nearly one third of hospitalizations after transplantation in the US [20]. Moreover, CVD is the predominant cause of death in kidney transplant recipients while death with functioning graft is the main cause of graft loss in the long term [2]. In the present study at least one CVE was noted among 13.7% of kidney transplant recipients and the most frequently diagnosed complication was myocardial infarction. The median time to first CV episode after last KT was almost 6 years.

Some authors reported that type of donor and, indirectly, quality of the graft may be a predictor of CVEs [3,4]. Babakry et al. observed that KT from a deceased donor increased more than twice the risk of CVEs in the 90-day postoperative period when compared to the transplantation from a living donor [21]. This association has not been confirmed by others in the long-term observation after renal transplantation [22]. In the present study, all CVEs which occurred within the first year after transplantation were excluded from the analysis. Notably, the median time to first CV episode as well as the structure of cardio- and cerebrovascular events did not differ between KDRI cohorts. Although we showed a significant difference in the incidence of CVEs between Q1 and Q3 we were not able to indicate such an association for the highest-KDRI (Q4) group. Similarly to other authors, we assumed at the beginning that low- and medium-quality kidneys would obtain comparable outcomes [23]. One of the possible explanations is that patients in Q4 were significantly older at transplantation than the rest of study participants and therefore their expected mortality rate, in particular from something other than a cardiovascular cause (mainly infections and malignancy), was greater and may mask the potential impact of organ quality in this group. Moreover, according to Huang et al., transplantation may temporarily attenuate the effect of age on cardiovascular morbidity and mortality [24]; thus, we may suspect that the quality of deceased donor kidneys may affect cardiovascular outcomes in elderly recipients not in the first years after KT, but rather in the long term. On the other hand, Pippias et al. [13] indicated that the difference in the 5-year survival probabilities between the two highest KDRI quartiles appeared to be narrowing in the last years, probably due to an improvement in the overall prevention, screening, and treatment in the highest KDRI category.

As expected, recipient age independently influenced the risk for post-transplant CVEs in the study group. Of note, Morales et al. [25] reported that the highest incidence of cardiovascular diseases at 5 years post-transplant was found among patients between 40 to 60 years of age. When we performed a subanalysis among kidney transplant recipients younger than 50 years at the transplantation, we observed a significant increasing trend for the occurrence of post-transplant cardiovascular complications along the consecutive KDRI quartiles. These results are consistent with those presented by Hernandez et al. [26] who showed that the receiving of a low-quality kidney seemed to significantly decrease patient survival among recipients aged 50 to 69 years but not in those aged 70 to 79 years. Similarly, Peters-Sengers et al. [27] reported that the association between the KDRI and graft failure was higher for younger recipients and the effect of the KDRI on graft survival significantly decreased with every 5-year increase in recipient age in this Dutch population. Therefore, a lower quality of kidneys from a deceased donor, those with a higher KDPI, may have a more pronounced effect in lower-risk recipients who are nondiabetics and in those with a younger age at transplantation, like in the study of Heaphy et al. [28]. Notably, when we evaluated the association between the KDRI and CVEs in a multivariate analysis, only recipient age, pretransplant ischemic heart disease, and post-transplant diabetes mellitus, but not KDRI value, independently influenced the risk of CVEs in a subset of recipients younger than 50 years. Based on the results of our study, we suggest that transplantation of high-KDRI kidneys to recipients before the age of 50, without comorbidities such as DM or CVD, should be considered carefully. On the other hand, older patients, particularly those with diabetes mellitus or a long-expected waiting time for kidney transplantation, could derive a long-term survival benefit receiving lower-quality kidneys than remaining on dialysis therapy, like it was presented in the studies by Pascual et al. [29] and Massie et al. [30].

The present study has some limitations. First of all, this is a single-center observational study; thus, caution is advised in the interpretation and extrapolation of our results for the whole European population. Furthermore, the analysis of grafts discarded during this follow-up period was beyond the scope of our study. Also, the retrospective nature of the study does not allow us to perform multivariate regression analysis of all traditional and non-traditional factors independently associated with CVEs after KT. Another limitation is a follow-up period shorter than 10 years. Such longer observation would be more reliable in analyzing the potential association between the quality of deceased-donor kidneys and cardiovascular outcomes, especially in elderly recipients. Moreover, this US-derived scoring system may not by ideal for the Polish donor cohort due to the very low percentage of donors with diabetes, the presence of anti-HCV antibodies, and DCD donors. Moreover, the donor cohort was exclusively Caucasian in this study. The algorithm proposed by Rao et al. includes a Black race coefficient. However, in recently published studies, removing donor race from the original KDRI did not change the predictive value of this model [31,32]. Nevertheless, when considering the possible association between the KDRI and long-term graft function in comparable years, it should be mentioned that the median KDRI in our study group was 1.092, compared with 1.05 for the US population from the analysis of Rao et al. [6] and 1.17 among kidney transplant recipients from the European cohort [10]. The Estimated Post-Transplant Survival (EPTS) score was implemented in the US as a recipient risk prediction tool for the outcome of patient survival after kidney transplantation [33]. The EPTS has not been evaluated in the Polish population so far. We will perform another analysis in the near future that takes into account the impact of both the KDRI and EPTS on kidney allocation in the Polish cohort. Finally, it should be emphasized that the use of a distinct KDPI or KDRI cut-off should be interpreted with caution, because the discriminatory power of the KDPI is modest (concordance index of 0.621).

## 5. Conclusions

In conclusion, the results of our study indicate that the KDRI is also a valuable prognostic tool for early and long-term outcomes after KT for European populations. Despite the KDRI not being used for allocation in Poland, we found that kidneys with a higher KDRI are allocated to recipients with a shorter life expectancy; however, this allocation resulted in a still quite acceptable long-term graft function. The median time to first CVE as well as the structure of cardio- and cerebrovascular events did not differ between KDRI cohorts. A significant increasing trend for the occurrence of post-transplant cardiovascular complications along the consecutive KDRI quartiles was observed among kidney transplant recipients younger than 50 years at the transplantation. Therefore, the lower quality of kidneys from a deceased donor, those with a higher KDPI, had a more pronounced effect in lower-risk recipients who are nondiabetics and with a younger age at transplantation. Transplantation of high-KDRI kidneys to recipients before the age of 50, without comorbidities such as DM or CVD, should be considered carefully.

## Figures and Tables

**Table 1:** Clinical characteristics of kidney transplant recipients divided into KDRI quartiles.

Parameter	KDRI Quartiles				*p*
	1 (*n* = 355)	2 (*n* = 356)	3 (*n* = 357)	4 (*n* = 352)	
Recipient characteristics at transplantation					
Age * [years]	45 (33–55) ^†††^ ^	46 (35–56) ^†††^	48 (37–57) ^†††^	53 (44–61)	<0.001
Sex [M/F]	225/130	201/155	218/139	211/141	0.30
BMI * [kg/m^2^]	24.1 (21.4–27.1) ^††^	23.8 (21.4–26.8) ^†††^	24.5 (21.5–27.3) ^†^	25.0 (22.3–27.8)	<0.01
Dialysis vintage * [months]	31 (17–54)	33 (18–56)	28 (18–48)	31 (18–48)	0.43
Hypertension [%]	310 (87.3)	313 (87.9)	320 (89.6)	312 (88.6)	0.72
Number of antihypertensive drugs * [*n*]	2 (1–3) ^#^	1 (1–2)	2 (1–3) ^#^	2 (1–3) ^#^	<0.05
IHD [*n* (%)]	35 (9.9) ^$^	37 (10.4) ^$^	49 (13.7)	57 (16.2)	<0.05
Pre-transplant CVEs [*n* (%)]	35 (9.9)	35 (9.8)	41 (11.8)	51 (14.5)	0.17
Diabetes [*n* (%)]	38 (10.7)	39 (11.0)	28 (7.8)	44 (12.5)	0.23
**Transplant procedure characteristics**					
Retransplant [*n* (%)]	62 (17.5) ^‡‡^	63 (17.7) ^‡‡^	35 (9.8)	47 (13.4)	<0.01
HLA class I mismatch	2.4 (2.3–2.5)	2.3 (2.2–2.4)	2.3 (2.2–2.4)	2.4 (2.3–2.5)	0.66
HLA class II mismatch	0.74 (0.67–0.80)	0.68 (0.62–0.75)	0.70 (0.63–0.77)	0.78 (0.71–0.85)	0.21
CIT [h]	17.7 (17.1–18.4) ^††^	18.6 (17.9–19.3)	18.5 (17.8–19.1)	19.3 (18.7–20.0)	<0.05
Induction therapy [*n* (%)]					
None	261 (73.5)	258 (72.5)	261 (73.1)	224 (63.6)	
IL-2RB	64 (18.0)	54 (15.2)	64 (17.9)	77 (21.9)	<0.05
ATG	30 (8.5)	44 (12.3)	32 (9.0)	51 (14.5)	
Initial immunosuppression [*n* (%)]					
CyA/Tc	197/157	144/212 ^^^	138/217 ^^^	118/245 ^^^	<0.001
AZA/MMF/mTORi	64/279/11	67/296/9	61/281/9	37/306/11	0.09
**Post-transplant complications**					
PTDM [*n* (%)]	85 (23.9) ^#^	60 (16.9)	91 (25.5) ^##^	89 (25.3) ^##^	<0.05
AR episode [*n* (%)]	64 (18.0)	50 (14.0)	52 (14.6)	64 (18.2)	0.28
CMV infection [*n* (%)]	34 (9.6)	40 (11.2)	29 (8.1)	46 (13.1)	0.16

Data presented as means with 95% confidence intervals, * medians with Q1–Q3 values, or frequencies. Statistics: ANOVA, Kruskal–Wallis test, or χ^2^ test; for between-group comparisons: Mann–Whitney U test or χ^2^ test. ^†^ *p* < 0.05, ^††^ *p* < 0.01, ^†††^ *p* < 0.001 for Q4 vs. other groups; ^ *p* < 0.05, ^^^ *p* < 0.001 for Q1 vs. other groups; ^#^ *p* < 0.05, ^##^ *p* < 0.01 for Q2 vs. the other groups; ^$^ *p*< 0.05 for Q1 and Q2 vs. Q4; ^‡‡^ *p* < 0.01 for Q1 and Q2 vs. Q3. BMI, body mass index; IHD, ischemic heart disease; CVEs, cardiovascular events; HLA, human leukocyte antigen; CIT, cold ischemia time; IL-2RB, interleukin 2 receptor blocker; ATG, antithymocyte globulin; CyA, cyclosporine A; Tc, tacrolimus; AZA, azathioprine; MMF, mycophenolate mofetil or sodium; mTORi, mammalian target of rapamycine inhibitor; PTDM, post-transplant diabetes mellitus; AR, acute rejection; CMV, cytomegalovirus.

**Table 2:** Early post-transplant and long-term kidney graft function in study groups based on KDRI quartiles.

Parameter	KDRI Quartiles				*p*
	1 (*n* = 355)	2 (*n* = 356)	3 (*n* = 357)	4 (*n* = 352)	
Early graft function					
IGF [%]	42.0	22.8	15.7	8.8	<0.001 *
SGF [%]	37.7	46.3	47.9	48.9	<0.05 *
DGF [%]	18.0	28.1	33.0	38.1	<0.001 *
PGN [%]	2.3	2.8	3.4	4.2	0.48
Follow-up eGFR [mL/min/1.73 m^2^]					
3 months ^1^	62.4 (49.2–77.5) *n* = 338	55.2 (44.0–64.6) *n* = 325	50.6 (40.0–61.8) *n* = 319	42.6 (31.7–53.2) *n* = 316	<0.001
12 months ^1^	65.0 (51.8–80.7) *n* = 327	57.1 (44.3–68.0) *n* = 318	50.5 (38.5–63.2) *n* = 314	44.7 (33.8–55.1) *n* = 300	<0.001
5 years ^1^	65.0 (48.5–78.3) *n* = 271	55.4 (43.2–68.8) *n* = 249	49.8 (35.1–60.7) *n* = 256	43.7 (33.6–54.7) *n* = 215	<0.001

Data presented as medians ^1^ with Q1–Q3 values or frequencies. Statistics: Kruskal–Wallis test or χ^2^ test. * *p* for trend. IGF, immediate graft function; SGF, slow graft function; DGF, delayed graft function; eGFR, estimated glomerular filtration rate.

**Table 3:** Univariate logistic regression analysis of risk factors for the occurrence of post-transplant CVEs in the whole study group and in the subset of recipients less than 50 years of age at transplantation.

Parameter	Whole Study Group			Recipients Aged <50 Years		
	OR (95% CI)	χ^2^	*p*	OR (95% CI)	χ^2^	***p***
Recipient age	1.04 (1.02–1.05)	31.6	<0.001	1.10 (1.07–1.14)	38.2	<0.001
BMI	1.05 (1.00–1.09)	4.8	<0.05	1.06 (1.00–1.13)	3.7	0.06
Number of antihypertensive drugs	1.04 (0.93–1.17)	0.52	0.47	1.02 (0.86–1.22)	0.06	0.81
IHD	2.42 (1.66–3.54)	19.1	<0.001	4.37 (1.98–9.65)	11.1	<0.001
Retransplantation	0.68 (0.42–1.09)	2.8	0.1	0.73 (0.39–1.39)	0.95	0.34
CIT	1.01 (0.99–1.04)	0.96	0.33	1.02 (0.99–1.06)	1.6	0.21
Induction therapy	0.71 (0.50–1.01)	3.8	0.06	0.78 (0.45–1.36)	0.8	0.38
PTDM	1.91 (1.36–2.70)	13.0	<0.001	2.14 (1.26–3.63)	7.4	<0.01
KDRI	1.29 (0.87–1.91)	1.5	0.22	2.32 (1.22–4.41)	6.6	<0.01

Data presented as OR with 95% CI. BMI, body mass index; IHD, ischemic heart disease; CIT, cold ischemia time; PTDM, post-transplant diabetes mellitus; KDRI, Kidney Donor Risk Index.

## Data Availability

The data presented in this study are available on request from the corresponding author.
